# Ventilation failure due to endotracheal tube T-connector defect

**DOI:** 10.4103/0019-5049.68382

**Published:** 2010

**Authors:** Chetna Shamshery, Ashish K Kannaujia, Shefali Gautam

**Affiliations:** Department of Anesthesia, Chhatrapati Shahuji Maharaj Medical University, Lucknow, Uttar Pradesh, India

Sir,

Despite the check steps or visual inspection for physical defect, incidences of device failures are commonly encountered. A 1-month-old male infant weighing 3.2 kg was presented for pyloromyotomy in the elective OT due to infantile hypertrophic pyloric stenosis. Apart from the lump in the upper abdomen, there were no significant other medical complaints. Inside the operation theatre, the monitors were connected and then the patient was premedicated and preoxygenated using Jackson Rees modification of Ayre’s T tube circuit. Induction was done with ketamine, and after checking for adequate chest expansion, succinylcholine was given. After relaxation, laryngoscopy was done and intubation performed using a 3-mm id endotracheal tube (ETT) under vision.

**Figure 1:**
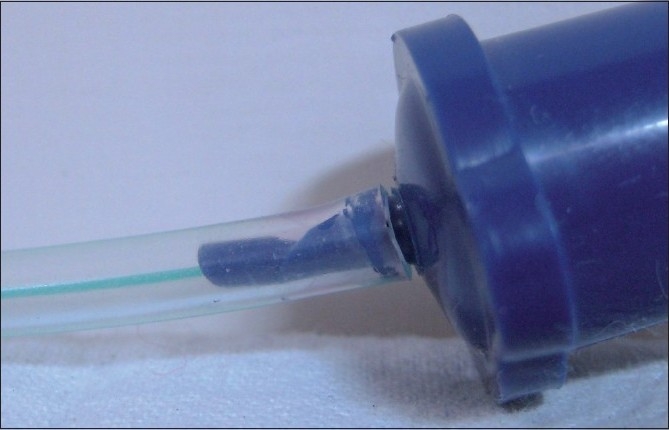
ETT showing deformed connector

On connecting the ETT to the circuit, the chest did not expand on ventilation, neither was there any air entry on auscultation. So, laryngoscopy was done and the position of the tube was assured; still the chest did not expand on ventilation. After this, the tube was taken out and without delay another tube of 3 mm id was connected. The baby was ventilated successfully, and on checking the previous tube, it was found that the T-connector of the ETT was obliterated. On inspection, it was found that this was a manufacturing defect [Figure 1] as the tube was new. There were no complications due to this delay of intubation.

ETTs are checked before intubation, but still device failures have been documented due to manufacturing defects,[1] e.g. cuff valve failure.[2] Other complications associated with the use of resterilised tubes,[3] breakage of part of the tube[4] or obliteration of the tube lumen by a foreign body, e.g. mucous plugs have also been documented. In our case, the 3-mm id ETT had obliteration in the T-connector, which caused ventilation failure. This was a manufacturing defect as the tube was neither being reused nor was resterilised. Usually on inspection, the obvious defects of the tubes are discovered but the defects which are visually not very perceptible are missed. This incidence signifies the importance of reviewing equipment defects for internal auditing purpose, so that complications could be avoided, because negligence could cost us a life.
